# Supplementary material for: Macrophage Migration Inhibitory Factor Suppresses Natural Killer Cell Response and Promotes Hypoimmunogenic Stem Cell Engraftment Following Spinal Cord Injury
Source: Biology (Basel). 2025 Jun 30;14(7):791. doi: 10.3390/biology14070791 (PMC12293055; doi:10.3390/biology14070791)
Supplement: Supplementary file 1 [file biology-14-00791-s001.zip › biology-3661238-supplementary.pdf]

Supplementary Materials

Supplementary Table S1. Guide RNA sequences for CRISPR mediated B2M and CIITA knockout

Gene Name	B2M	CIITA
Transcript ID	ENST648006.3	ENST00000324288.12
guide RNA 1	GGCCGAGAUGUCUCGCUCCG	CACAGCUGAGCCCCCACUG
guide RNA 2	ACUCACGCUGGAUAGCCUCC	GGCUCCUGGUUGAACAGCGC
guide RNA 3	CGGAGCGAGAGAGCACAGCG	CCCCUAACAUACUGGGAAUC
Forward primer	ACAGCAAACTCACCCAGTCTAG	TGAGAGCTTGGGGTCCCTTA
Reverse primer	CCAGTCTAAGGGAAGCAGAGC	CTGAGGCATGTTCTCTGCCA
Sequencing primer	AAACTCACCCAGTCTAGTGC	GGTAGGGGCTTGGAGCTAAC

Supplementary Table S2. Primer sequences for qPCR

Gene Name	Forward	Reverse
GAPDH	AATCCCATCACCATCTTCCAG	AAATGAGCCCCAGCCTTC
MIF	GTTCATCGTAAACACCAACGTG	GAAGGCCATGAGCTGGTC

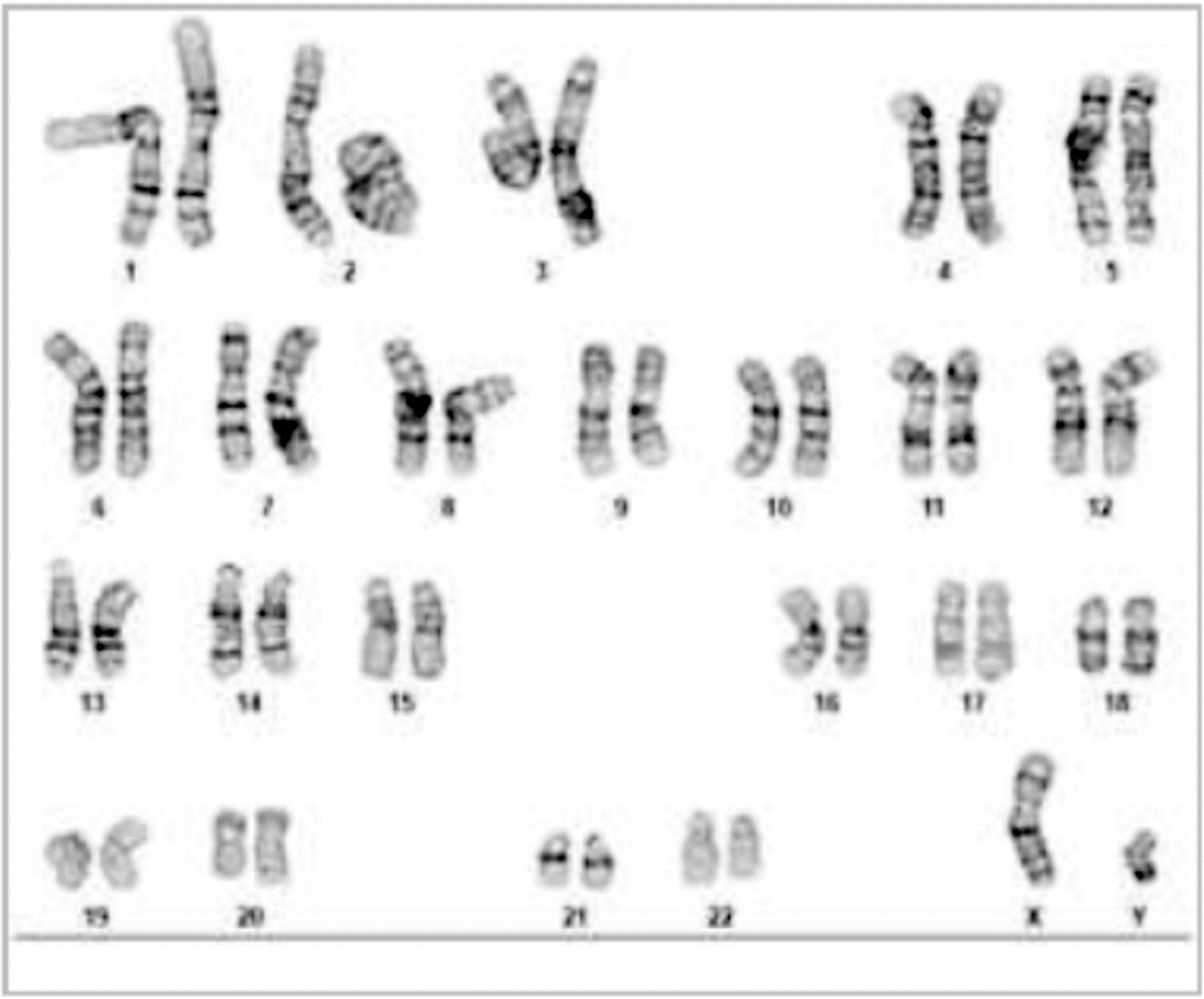

Supplementary Figure S1. NCL2-GFP maintained a normal karyotype. The parental cell line of this study, NCL2-GFP has maintained a normal karyotype.
